# Impact of Whole-Fruit Storage Conditions on the Quality of Minimally Processed Pears

**DOI:** 10.3390/plants14142108

**Published:** 2025-07-09

**Authors:** Vanessa Cuozzo, Eva Torres, Yanina Pariani, Ana Cecilia Silveira

**Affiliations:** Poscosecha de Frutas y Hortalizas, Dpto. de Producción Vegetal, Facultad de Agronomía, Udelar. Avda. Garzón 780, Montevideo CP 12900, Uruguay

**Keywords:** pear, minimally processed, controlled atmosphere storage, functional quality, polyphenol oxidase activity, flesh firmness

## Abstract

The shelf life of minimally processed fresh (MPF) pears is affected by raw material characteristics and production factors. This study evaluated the effect of raw material storage (3 months in regular atmosphere [RA], 3 and 6 months in controlled atmosphere [CA]) on the organoleptic and functional quality of MPF pears packaged in polypropylene (PP) and low-density polyethylene (LDPE) for 0, 10, and 15 days at 0 °C. Wedges from 3-month CA showed the lowest respiratory activity (about 8.31 mg CO_2_ kg^−1^ h^−1^), and those from 6-mounth CA maintained higher firmness after 15 days. Lightness decreased during storage, less so in harvest samples, which also showed less browning. Nevertheless, polyphenol oxidase (PPO) activity increased fivefold after 15 days. Total polyphenol content decreased by about 50% during storage. Wedges in PP packaging exhibited higher total antioxidant capacity (TAC) measured by DPPH than those in LDPE (15.55 and 13.77 mg EAA 100 g^−1^ FW, respectively). In both, the contents were reduced after 15 days (15–38%). No differences in TAC were observed in the FRAP assay, where values remained unchanged. Significant correlations between PPO activity, TAC, and color variables suggest ongoing oxidative processes. In contrast to the effect of raw material storage, the type of packaging did not significantly affect any of the measured variables.

## 1. Introduction

Minimally processed fresh (MPF) products have gained popularity due to their convenience and ready-to-eat format. However, the processing steps involved, such as peeling, cutting, washing, and sanitizing, cause mechanical stress and tissue damage, which accelerate respiration, enhance enzymatic activity, and promote oxidative degradation, leading to a significantly shorter shelf life compared to whole produce [1].

Pears (*Pyrus communis* L.) are widely consumed for their organoleptic and nutritional qualities and are suitable for minimally processing. Nevertheless, in MPF pears, two main challenges limit the preservation of quality, which are flesh softening and enzymatic browning, both of which are closely linked to tissue disruption and oxidative reactions [2]. Flesh softening in MPF products, like in ripening, is promoted by ethylene. This hormone increases considerably in MPF fruit, mainly because it is linked to stress processes. Therefore, the mechanical stress caused by processing itself triggers an increase in its biosynthesis. Ethylene induces the expression of genes encoding hydrolytic enzymes, such as polygalacturonase, pectin methyl esterases, cellulase, and pectinase, which degrade cell walls. Additionally, other processes, such as water loss, may reduce turgor pressure, further affecting firmness [3,4,5]. Enzymatic browning is another major contributor to quality loss in MPF fruit. This phenomenon primarily occurs when the enzymes polyphenol oxidase (PPO) and peroxidase (POD) come into contact with phenolic compounds, catalyzing their oxidation into o-quinones, which subsequently polymerize to form brown pigments known as melanins [6,7,8]. Under normal conditions, enzymes and their substrates are compartmentalized within different cellular structures. For instance, PPO is located in the thylakoids of chloroplasts, whereas phenolic compounds are stored in the vacuoles. However, during minimal processing, this compartmentalization is disrupted, leading to enzymatic browning reactions. A similar situation can occur in whole fruit during advanced ripening or senescence due to the loss of membrane integrity and selective permeability [9]. As a result, enzymatic browning is triggered, which not only affects the visual appearance of the product but also compromises its nutritional and functional quality. This decline is mainly due to the loss of antioxidant phenolic compounds, which are no longer available to neutralize reactive oxygen species (ROS), thus reducing the overall antioxidant capacity of the product [10,11].

Different aspects, including genotype, fruit ripeness, pre- and postharvest treatments, and storage conditions of raw materials, can influence MPF quality [12,13,14]. These factors modulate the physiological status of the fruit—such as antioxidant levels and enzymatic activity—which in turn affect its ability to tolerate wounding stress. Understanding their impact on product quality is crucial for maintaining quality and extending its shelf life, particularly since consumers expect consistent quality throughout the year. Among these factors, one of the most relevant is adjusting the storage conditions of the raw materials, especially since their production is, in most cases, seasonal. Additionally, the storage conditions of the raw materials used in MPF production can significantly impact the organoleptic and functional quality of the final product. Several studies have shown that the storage conditions of whole pear fruit—such as temperature, oxygen (O_2_) levels, and storage duration—can significantly influence physiological parameters such as respiration rate and ripening behavior, as well as antioxidant properties and overall quality. In this sense, cold storage—particularly under controlled atmosphere (CA) conditions—can effectively delay ripening, reduce respiration rate, and preserve antioxidant compounds, thereby helping to maintain product quality during storage [15,16,17]. However, extended storage may also lead to partial senescence or metabolic downregulation, potentially reducing the fruit’s responsiveness to subsequent processing. Furthermore, MPF products are often stored under modified atmosphere (MA) conditions. MA involves adjusting the gas composition—mainly O_2_, CO_2_, and N_2_—to reduce enzymatic activity and microbial growth [18,19]. Its effectiveness depends on selecting suitable packaging materials with appropriate gas permeability (mainly O_2_ and water vapor) to maintain internal conditions favorable for preserving product quality. Excess or insufficient gas exchange may accelerate deterioration or fail to adequately control enzymatic browning [15,16].

This study aimed to evaluate the effects of the preservation conditions (including storage duration and atmosphere) of the raw material, as well as packaging type and refrigerated storage time, on the physicochemical, enzymatic, functional, and microbiological quality of fresh-cut pears.

## 2. Results and Discussion

### 2.1. Respiration Rate

**Figure 1:**
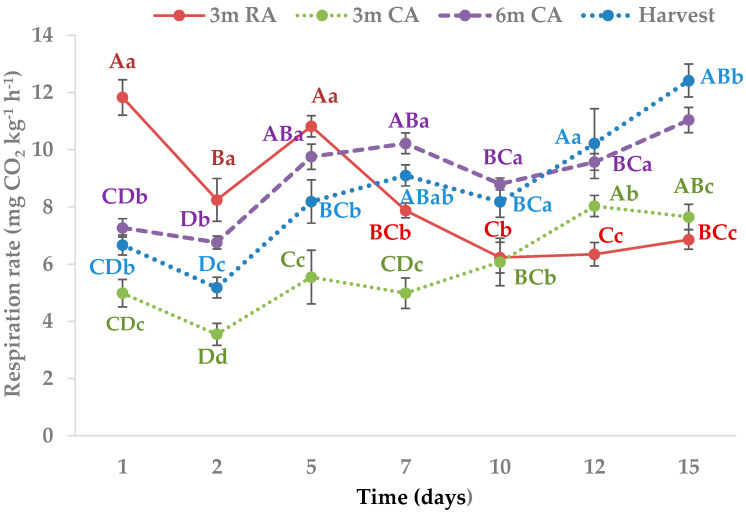
Respiration rate of pear wedges from different raw material origins (RA: regular atmosphere; CA: controlled atmosphere). Values are means (n = 4) ± standard error of the mean. Uppercase letters indicate differences among raw material origins in each moment, and lowercase letters indicate differences over storage time in each raw material origin (Tukey’s test, *p* < 0.05).

The respiration of the wedges showed a higher initial value than that measured on day 2, regardless of the origin of the raw material (Figure 1). This behavior has been widely reported in MPF products and is explained by the stress experienced by plant tissues due to the different operations they undergo during processing [20].

The respiration patterns over time in wedges from harvested raw material and those preserved for 3 and 6 months in CA were very similar, showing an increase between days 10 and 15 of refrigerated storage.

The most noticeable differences during storage were observed between days 1 and 5, where pieces from pears stored in regular atmosphere (RA) exhibited a respiratory rate that was 10 to 58% higher than that of pears from the other storage conditions. Until day 7, the respiratory rate of wedges made from 3 months CA pears remained considerably lower than that of the other treatments. However, between days 10 and 15, their behavior was practically indistinguishable from that of 3-month RA pears. The respiratory activity of MPF pears from harvested fruit and from 6-month CA storage was 58 to 75% higher, with no notable differences between them.

The measured respiratory rate was similar to that the reported by other authors. Gorny et al. [21] mentioned that the respiratory rate of MPF William’s pears during the first 3 days of storage at 0 °C ranged between 10.78 and 19.02 mg CO_2_ kg^−1^ h^−1^, which means the values measured in this study are close to the lower limit of that range. Additionally, Mattheis [15] found that pears stored under CA conditions had a significantly lower respiration rate, which is consistent with the results observed in this study.

### 2.2. Atmosphere Composition

After 15 days of wedge storage, O_2_ concentrations reached 15.77% and 16.5% in polypropylene (PP) and low-density polyethylene (LDPE) packages, respectively. CO_2_ levels at the end of the storage period ranged from 6.17% to 8.47% in PP and from 3.9% to 5.6% in LDPE. The recommended storage conditions for MPF pears typically range between 0.5% and 2.5% O_2_ and 2% to 10% CO_2_, concentrations known to delay enzymatic browning and microbial growth, thereby preserving product quality [22]. Previous studies have identified atmospheres with high CO_2_ levels, such as 2% O_2_ + 10% CO_2_, as optimal for preserving Conference MPF pears at 4°C for up to 12 days [23]. Similarly, Wang et al. [24] reported that 10% CO_2_ combined with 11% O_2_ significantly reduced the respiration rate of MPF pears, attributed to changes in enzyme activity and expression related to energy metabolism. Oguz-Korkut et al. [25] observed a 3- to 4-fold reduction in the respiration rate of Deveci MPF pears stored at 4°C under 4% O_2_ and 5% CO_2_ for 8 days.

Therefore, while the CO_2_ concentrations observed in this study fell within a range suitable for respiration control, the elevated O_2_ levels highlight a key limitation of the selected packaging materials, ultimately rendering them unsuitable for maintaining optimal storage conditions.

### 2.3. Flesh Firmness

**Figure 2:**
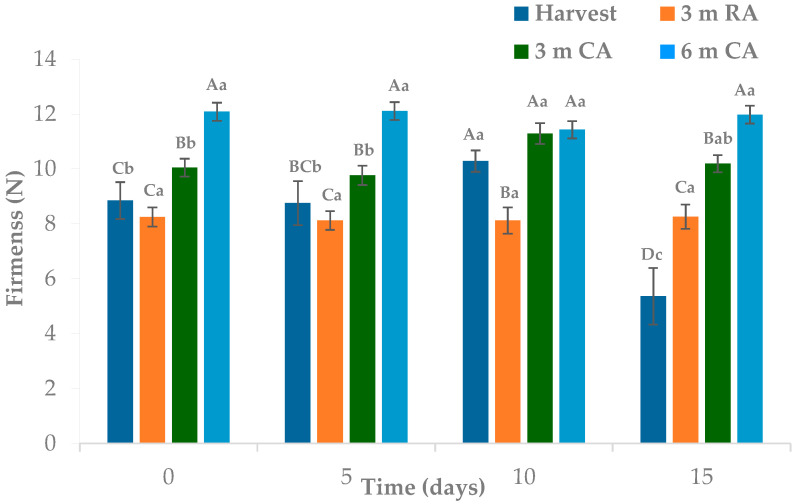
Flesh firmness of pear wedges from different raw material origins (RA: regular atmosphere; CA: controlled atmosphere). Values are means (n = 18) ± standard error of the mean. Uppercase letters indicate differences between raw material origins, in each storage time, and lowercase letters indicate differences over storage time (Tukey’s test, *p* < 0.05).

The storage conditions of the raw material and the duration of MPF storage significantly affected wedge firmness (Figure 2). In contrast, no significant differences were observed between packaging materials. Firmness was best preserved in samples prepared from fruit previously stored under CA conditions, which allowed for retention of this attribute for up to 6 months.

Wedges prepared from raw material stored in CA for 6 months were 15–30% firmer during the first 10 days of MPF storage. After 15 days, the differences became even more pronounced, ranging from 15% to 55%. As demonstrated in pears and other fruits, CA storage is a well-established technology for preserving firmness and overall quality over extended storage periods [21,26]. No substantial differences were observed among the remaining raw material storage treatments during the first 10 days. However, by day 15, wedges prepared from freshly harvested raw material showed a marked reduction in firmness, followed by those made from raw material stored in RA for 3 months. This finding is consistent with previous studies indicating that RA storage typically results in more rapid softening compared to CA storage [26,27].

Over time, firmness was generally maintained across treatments during the 15-day MPF storage period, except for wedges derived from freshly harvested raw material. This observation supports findings from recent studies showing that raw material storage conditions play a key role in the texture retention of minimally processed fruits [28].

### 2.4. Color

The lightness (L*) of pear wedges varied between treatments at each analysis time, as shown in Table 1. Up to 10 days of storage at 5 °C, the higher L* value corresponded to wedges from freshly harvested fruit and from fruit stored in CA for 3 and 6 months. However, after 15 days, wedges from freshly harvested fruit showed the lowest L*. This trend was consistent across both packaging materials. Only wedges from freshly harvested fruit exhibited significant changes in L* over time, with a marked decrease after 15 days. In the other treatments, storage time had no relevant effect on the L* parameter. These findings are in line with those of Akbari et al. [29], who reported similar behavior in fresh-cut pear cv. Shahmive.

At the beginning of storage, the hue or color tone (h*_ab_) values of wedges processed immediately after harvest ranged from 90 to 94, indicating a greenish-yellow hue typical of less ripe fruit. In contrast, wedges made from fruit previously stored in CA conditions showed a more yellow coloration. During storage, h*_ab_ values in wedges from freshly harvested fruit remained stable, while those from the other treatments progressively decreased, shifting toward more orange (80–84) and reddish tones (below 75). The emergence of orange and reddish hues after 5 days is associated with enzymatic browning processes.

**Figure 3:**
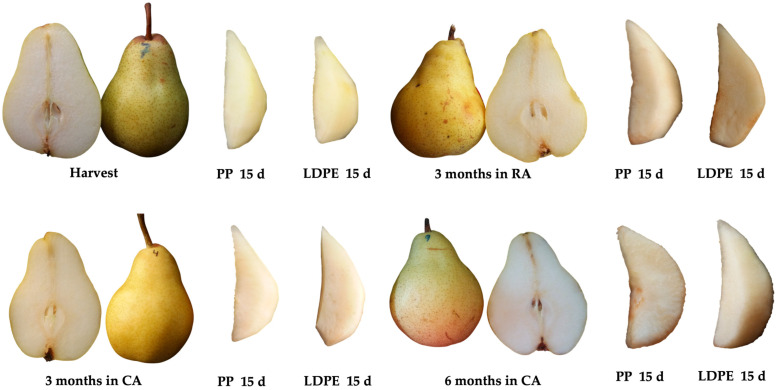
Whole and pear wedges from different raw material origins (RA: regular atmosphere; CA: controlled atmosphere) packaged in polypropylene (PP) and low-density polyethylene (LDPE) after 15 days.

Overall, significantly higher chroma or saturation (C*) values were observed in wedges made from raw material stored in CA conditions compared to those processed immediately after harvested or preserved in RA. This difference was particularly pronounced in wedges stored for 3 months in CA conditions. In contrast, the samples processed at harvest exhibited the lowest values across all evaluated moment, indicating lower color intensity from the outset. The reduced saturation seen in the harvest samples may be attributed to their greater susceptibility to oxidative and degradative processes, which negatively affect color. Additionally, packaging in PP was found to preserve color better than packaging in LDPE (Figure 3). Similarly, Sathya et al. [30] reported reductions in the L* and h_*ab_ values in pears, both coated and uncoated, during 15 days of storage at 4 °C. According to their findings, color changes from yellow to brown occurred within one day in the control samples and within three days in the coated ones.

Color differences, assessed through ΔE revealed that wedges from freshly harvested fruit underwent minimal color change after 5 days but exhibited highly noticeable differences by day 15 (10.25 and 16.01 for PP and LDPE packaging, respectively). In wedges from fruit stored for 3 months in a regular atmosphere (RA), the ΔE values remained around 3. Wedges from 3 months CA storage showed slightly higher differences (~3.5). The smallest color changes were found in wedges from fruit stored in CA for 6 months, with ΔE values between 1.5 and 2.96—indicating barely perceptible changes to consumers [31].

These results are in line with those of Allegra et al. [32], who reported an increase in BI over time in MPF Coscia and Abate Fetel pears, with the highest values observed after 12 days at 4 °C, especially in control samples. The decrease in L* and the shift toward more orange and reddish hues in the pears have been linked to enzymatic browning processes, which are often associated with PPO activity and the degradation of TP. These factors may contribute to the observed color changes [33,34]. In fact, in the present study, PPO activity sharply increased after 10–15 days of storage, especially in wedges processed from freshly harvested fruit, which also exhibited the most pronounced reduction in the L* and h_*ab_ values. The accumulation of oxidative stress during cold storage, combined with the higher O_2_ permeability of the packaging materials—particularly LDPE—likely enhanced enzyme activation and pigment oxidation. Moreover, these enzymatic reactions are favored by membrane deterioration, a typical outcome of chilling stress or senescence processes in minimally processed fruit [35]. Interestingly, despite minimal processing causing immediate tissue disruption and contact between PPO and existing phenolic substrates, significant browning was not observed until approximately 12 days of storage. This delayed onset suggests that browning is not solely dependent on PPO activity. According to Cao et al. [36], tissue wounding upregulates phenylalanine ammonia-lyase (PAL) at transcriptional and enzymatic levels, enhancing de novo phenolic biosynthesis. This increased phenolic substrate availability over time fuels PPO-mediated oxidation, explaining the progressive browning observed. Therefore, the visual deterioration in our samples may result from the synergistic effect of increased PPO activity, reduced antioxidant defenses, and PAL-induced accumulation of phenolic compounds during storage.

### 2.5. Polyphenol Oxidase Activity

**Figure 4:**
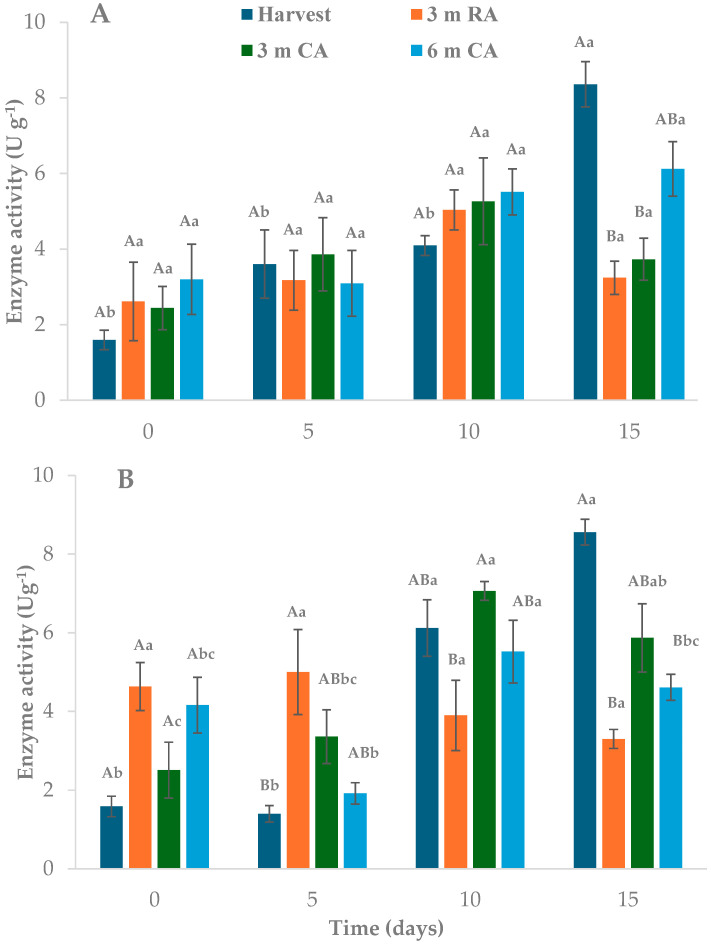
Polyphenol oxidase activity of pear wedges from different raw material origins (RA: regular atmosphere; CA: controlled atmosphere) and packaging materials ((**A**): PP; (**B**): LDPE). Values are means (n = 3) ± standard error of the mean. Uppercase letters indicate differences between raw material origins, in each storage time, and lowercase letters indicate differences over storage time within each packaging type (Tukey’s test, *p* < 0.05).

Given its role in browning, PPO activity was monitored throughout refrigerated storage to better understand the enzymatic response in pear wedges derived from different raw material origins. Enzyme activity measured in wedges stored in PP packaging showed no significant differences between treatments during the first 10 days (Figure 4A). However, by day 15, the highest activity was observed in wedges from freshly harvested fruit (8.36 U g^−1^), while the lowest values were recorded in those from fruit stored for 3 months under RA and CA conditions (3.24 and 3.73 U g^−1^, respectively). After 15 days, enzyme activity in wedges processed immediately after harvest was five times higher than at the beginning. Wedges preserved in LDPE exhibited a similar trend, with differences due to raw material origin becoming evident after 15 days (Figure 4B). In this case, wedges made from freshly harvested pears showed the highest PPO activity, followed by those stored for 3 months in CA. Over time, enzyme activity increased in wedges from freshly harvested pears, particularly after 10 days at 5 °C. Although the overall trends were comparable between the packaging materials, PPO activity levels tended to be slightly higher in LDPE than in PP, suggesting a potential influence of packaging permeability on oxidative enzymatic response.

The fruit’s physiological condition at the time of minimal processing influenced its enzymatic response. In freshly harvested fruit, wounding stress likely triggered a strong oxidative response, rapidly inducing PPO activity. In contrast, fruit that underwent extended cold storage—particularly under CA conditions—may have experienced metabolic adaptation or progressed into advanced stages of senescence. These physiological changes are known to reduce the tissue’s ability to activate defense mechanisms in response to stress [9,37]. Aged tissues often exhibit attenuated wound-induced phenylpropanoid biosynthesis and enzyme activity, which is associated with factors such as impaired signal transduction. Additionally, the availability of O_2_, which varies depending on the packaging material, may also influence PPO activity. Since PPO requires molecular O_2_ to carry out its catalytic activity, the higher permeability of LDPE could have favored the enzyme’s action compared to PP. Therefore, the observed patterns in PPO activity may result from the combined effect of tissue responsiveness, O_2_ availability, and the presence of phenolic substrates.

No studies were found that specifically address the effect of raw material storage condition on PPO activity in MPF. However, several works have evaluated the evolution of PPO activity during storage MPF fruit. In most of these studies, PPO activity followed patterns similar to those observed in wedges made from freshly harvested pears. For instance, Akbari et al. [29] reported that PPO activity increased over time in fresh-cut pear cv. Shahmiv. In untreated samples (without antioxidant treatments), PPO activity reached 123.2% of the initial value after 7 days of storage. Similarly, Ali et al. [38] noted that enzymatic browning in fresh-cut apples was consistently accompanied by increased activity of PPO and POD enzymes, which catalyze the conversion of phenolic compounds to quinones. Chen et al. [17], investigating the effect of flavonoids on browning control in fresh-cut Yuluxiang pear, observed that PPO activity increased in both untreated control samples and in those treated with 0.5 mg mL^−1^ flavonoids during the first four days of storage at 4 °C, before subsequently decreasing.

### 2.6. Total Polyphenol Contents and Antioxidant Activity

**Figure 5:**
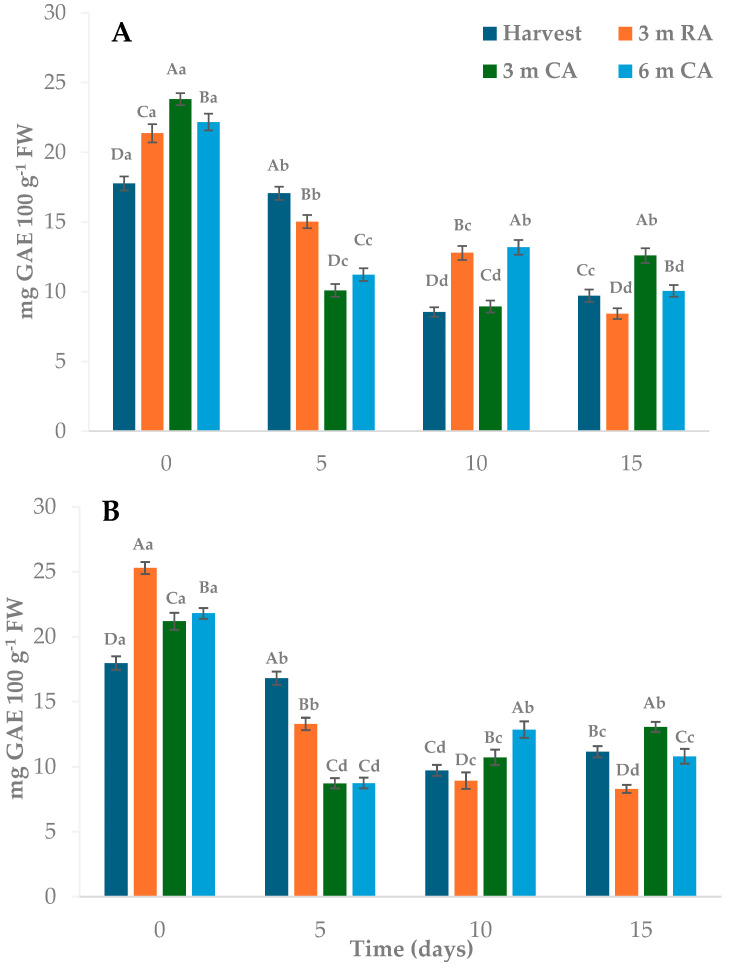
Total polyphenol content of pear wedges from different raw material origins (RA: regular atmosphere; CA: controlled atmosphere) and packaging materials ((**A**): PP; (**B**): LDPE). Values are means (n = 3) ± standard error of the mean. Uppercase letters indicate differences between raw material origins, in each storage time, and lowercase letters indicate differences over storage time within each packaging type (Tukey’s test, *p* < 0.05).

The storage conditions of the raw material influenced the total polyphenol content (TP). In both packaging types, wedges made from freshly harvested fruit initially showed TP values between 15% and 29% lower than those measured in wedges from other treatments (Figure 5A,B). These initial differences suggest that pre-processing storage can significantly affect phenolic compound levels. Generally, TP tends to decrease during storage, although the extent of this reduction varies depending on the specific storage parameters applied; for instance, in a study, pear cv. Patharnakh was stored at 0–1 °C under RA for 70 days, and a linear decrease was observed, with the TP content reduced to approximately half its initial level by the end of the storage period [39]. By contrast, CA storage tends to better preserve TP, mainly due to lower oxidative activity under reduced O_2_ conditions. CA is widely employed to extend shelf life and mitigate physiological disorders [25,28]. Therefore, it was expected that raw material stored under CA conditions (for 3 and 6 months) would retain higher TP levels—or at least levels comparable to those in pears harvested at maturity or stored for shorter durations under RA. Over time, the TP values declined across all treatments. After 5 days, and contrary to the initial findings, wedges from freshly harvested pears exhibited the highest TP retention, with losses ranging from 4% to 7%. In contrast, reductions in other treatments ranged from 30% to 60%. Between days 10 and 15, the TP content remained relatively stable, with the highest levels observed in wedges prepared from fruit stored for 3 and 6 months under CA. In our study, TPC decreased progressively during storage, reaching approximately 50% reduction after 15 days, regardless of treatment. This trend is consistent with previous reports on MPF products. For instance, Men et al. [40] found an almost 50 percent reduction in fresh-cut pear cv. Dangshansu without antioxidant treatment. Similar decreases were observed in pineapple slices and carambola under refrigerated storage, although the extent of reduction varied with the type of treatment applied [27,41].

No substantial differences were observed between packaging materials, and no significant effect of the selected plastics on polyphenol preservation was detected, likely due to their limited ability to regulate internal gas composition, particularly O_2_ levels. The observed changes in TP content may be associated with enzymatic browning processes involving PPO activity and color degradation. These potential relationships will be further examined in the correlation analysis section.

**Figure 6:**
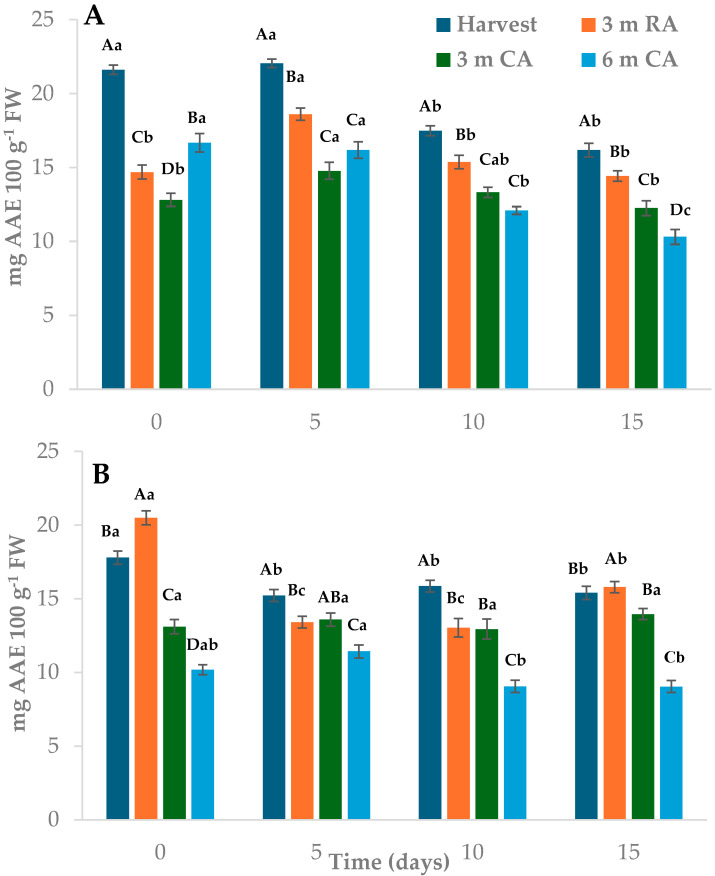
Total antioxidant capacity, measured by DPPH method, of pear wedges from different raw material origins (RA: regular atmosphere; CA: controlled atmosphere) and packaging materials ((**A**): PP; (**B**): LDPE). Values are means (n = 3) ± standard error of the mean. Uppercase letters indicate differences between raw material origins, in each storage time, and lowercase letters indicate differences over storage time within each packaging type (Tukey’s test, *p* < 0.05).

Total antioxidant capacity (TAC) measured by DPPH varied depending on the raw material preservation treatment, storage time of the MPF, and packaging material (Figure 6). Wedges stored in PP packaging exhibited higher TAC values than those in LDPE, with averages of 15.55 and 13.77 mg EAA 100 g^−1^ FW, respectively. At the beginning of storage, wedges made from freshly harvested fruit and packed in PP showed TAC values 23–41% higher than those from other treatments (Figure 6A). These values remained nearly unchanged until day 5, followed by a gradual decline. After 15 days, wedges from fruit stored for 6 months under CA showed a 15% decrease in TAC compared to day 10, and a 38% reduction relative to their initial values. For the remaining treatments, TAC values did not differ significantly between days 10 and 15. In LDPE-packaged wedges, the highest TAC was found in those made from pears stored for 3 months under RA, with values 13–50% higher than the other treatments’ (Figure 6B). A decline in TAC was observed after 5 days of storage, although the levels remained relatively stable up to day 15. Across all evaluation times, wedges from fruit stored for 6 months under CA consistently exhibited TAC values 40.50% lower than those from other treatments.

**Figure 7:**
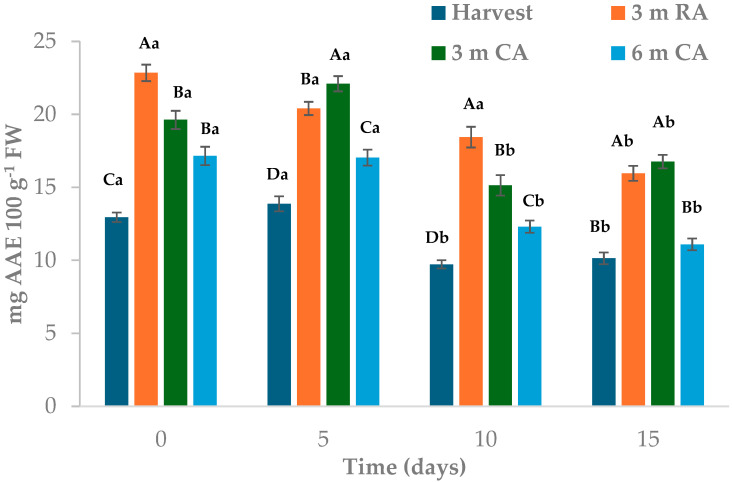
Total antioxidant capacity, measured by FRAP method, of pear wedges from different raw material origins (RA: regular atmosphere; CA: controlled atmosphere) Values are means (n = 3) ± standard error of the mean. Uppercase letters indicate differences between raw material origins, in each storage time, and lowercase letters indicate differences over storage time (Tukey’s test, *p* < 0.05).

When TAC was assessed using FRAP, the type of packaging material had no significant influence. Wedges from freshly harvested fruit showed values between 27% and 50% lower than those from other treatments (Figure 7). In most cases, the highest values were observed in wedges prepared from fruit stored for 3 months under RA or CA. Throughout storage, TAC remained relatively stable during the first 5 days in most treatments, followed by a reduction of 15% to 30% by day 10. These values remained largely unchanged through to day 15. By the end of storage, wedges from freshly harvested fruit and those stored for 6 months under CA exhibited TAC levels 35–45% lower than the other treatments, with no significant differences between them.

The observed reduction in TAC during refrigerated storage may be related to oxidative processes triggered by mechanical stress and increased metabolic activity in MPF pears. Processing steps such as peeling and cutting are known to promote ROS production, which, in turn, contributes to the degradation of polyphenols and vitamin C—key contributors to antioxidant capacity [42].

The TAC decline observed in this study aligns with such findings, particularly in samples made from fruit stored under prolonged CA or RA. A similar trend was also reported by Sharma and Rao [43] in MPF Asian pears. In their study, TAC (assessed by DPPH) decreased after 2 days of storage at 4 °C and remained stable thereafter in most treatments. The authors also reported a positive correlation between TAC and polyphenol content, highlighting the interconnected behavior of these antioxidant components.

### 2.7. Microbiological Growth

The type of packaging used did not significantly influence the growth of any of the microbial groups evaluated. As expected, the microbial levels were affected by the storage time of the MPF product. In the case of Enterobacteriaceae, the counts remained below 1 log CFU g^−1^ up to day 10. After that, the levels increased to 1.1 log CFU g^−1^ and reached 1.6 log CFU g^−1^ by day 15. For mesophilic aerobes, a significant increase was observed at 10 and 15 days of storage, particularly in wedges prepared from freshly harvested fruit and from fruit stored for 6 months under CA (average values of 2.24 and 2.85 log CFU g^−1^, respectively). Regarding psychotropic microorganisms, the most pronounced growth was recorded at the end of storage, with average counts of 1.78, 3.04, and 5.25 log CFU g^−1^ in wedges prepared from fruit stored for 3 m under RA, 3 m under CA, and 6 m under CA, respectively. Yeast and mold counts remained below 1 log CFU g^−1^ throughout the 15-day storage period.

In general, MPF fruits, due to their compositional characteristics—particularly their high organic acid content—do not exhibit elevated microbial growth, as observed in other substrates, such as vegetables. In this regard, Soliva-Fortuny et al. [44] noted that the high organic acid content in fruits contributes to a low pH, which acts as a natural barrier to microbial proliferation. However, this protection is susceptible to biochemical changes that occur during the storage of freshly cut fruit, which could alter the product’s pH. In contrast to our results, Siddiq et al. [45] reported substantially higher microbial levels in MPF D’Anjou pears stored at 4 °C for 21 days, attributing this outcome to the greater effectiveness of MAP conditions in reducing microbial growth. In our study, the limited atmospheric modification achieved with PP and LDPE films likely did not contribute significantly to microbial control. Instead, the low microbial growth observed may be more closely related to the characteristics of the raw material and proper handling, which may have helped maintain microbial stability despite the absence of effective MAP.

### 2.8. Correlation Analysis of Biochemical, Physical, and Color Parameters

**Figure 8:**
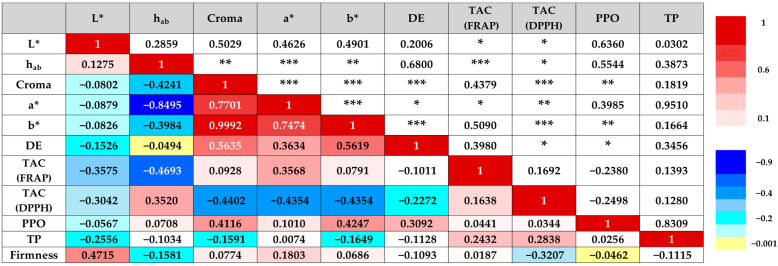
Pearson’s correlation analysis between physicochemical parameters of pear wedges. Significant correlations are indicated by asterisks *p* < 0.05 (*), *p* < 0.01 (**), *p* < 0.001 (***).

The correlations identified between PPO activity, CAT, and color variables indicate that, as expected, oxidative processes are involved in the color changes observed during the storage of MPF pears (Figure 8).

PPO showed positive correlations with C* (r = 0.4116; *p* = 0.0003), b* (r = 0.4247; *p* = 0.0002), and ΔE (r = 0.3092; *p* = 0.0082), suggesting that its activity is associated with increased color saturation and greater total deviation from the initial color, likely because of enzymatic browning. TAC, meanwhile, exhibited significant correlations with multiple color parameters, although with differing trends depending on the method used. Using the FRAP method, a negative correlation was found with the L* parameter (r = −0.3575; *p* = 0.002) and h_ab_* (r = −0.4693; *p* < 0.0001), along with a positive correlation with a* (r = 0.3568; *p* = 0.0021), indicating that reduced antioxidant capacity is linked to product darkening and a shift toward reddish tones. However, in both cases, lower antioxidant capacity was associated with visible color changes attributed to oxidative processes. DPPH analysis consistently showed negative correlations with L*, C*, a*, and b* (r values between −0.30 and −0.44), and a positive correlation with h_ab_* (r = 0.3520; *p* = 0.0024). These results suggest that the decline in antioxidant capacity is associated with product darkening and a reddish shift due to enzymatic browning involving phenolic compounds.

In addition, fruit firmness showed a significant positive correlation with L* (r = 0.4715; *p* < 0.0001), indicating that firmer fruits tended to be lighter in color. This finding suggests that firmness loss, characteristic of softening during storage, is also associated with tissue darkening. In other words, the deterioration process entails structural and compositional degradation, affecting the product’s texture, composition, and visual appearance.

Although Silva and Sulaiman [46] reported that the TP content in pear fruit is relevant to enzymatic browning, no significant correlations were found between TP and any of the variables analyzed in our study. Ercoli et al. [47] reported similar results, with a positive correlation between PPO and ΔE, suggesting that this enzyme is a major contributor to browning in apple pulp. Although this may seem self-evident, PPO is not always associated with browning [48]. For instance, in studies on MPF potatoes, browning was found to be unrelated to PPO activity or phenolic content [49].

## 3. Materials and Methods

### 3.1. Raw Material

The experiment was conducted using William’s pears manually harvested at commercial maturity, according to the producer’s criteria, from a commercial orchard located in the locality of Melilla (34°46′59″ S, 56°20′38″ W), Montevideo Department, Uruguay. Fruits were selected based on uniformity of size and absence of visible defects.

After harvest, the pears were stored under controlled atmosphere (CA; 0–1 °C, 97% RH, 3% CO_2_ and 3% O_2_) and regular atmosphere (RA; 0–1 °C, 97% RH) conditions for 0 (harvest), 3, and 6 months.

The day before processing, the fruits were washed with tap water at room temperature, drained for 5 min, and stored at 0 °C and 95% RH.

### 3.2. Minimally Processed

Fruits showing any defects, such as bruises, wounds, or physiological disorders, were discarded. Processing was performed in a clean, sanitized processing room maintained at 7 °C. The fruits were first peeled using a sharp stainless-steel knife and then cut into eight wedges with a domestic fruit cutter.

Immediately after cutting, the pear wedges were placed in a container with tap water at approximately 4 °C to remove cellular fluids, reduce enzymatic browning, and lower their temperature. The wedges were disinfected with 150 ppm sodium hypochlorite (NaClO, 10% active chlorine; Droguería Paysandú, Montevideo, Uruguay) at 4 °C for 2 min, and then rinsed with tap water.

Next, they were immersed in a 1% ascorbic acid solution (C_6_H_8_O_6_; analytical grade, DIU, Montevideo, Uruguay) for 2 min to further prevent browning. After that, excess surface water was removed using a manual vegetable centrifuge (Ilko, Santiago, Chile).

Packaging was carried out in heat-sealed 15 × 7 cm bags made of polypropylene (PP, ~150–200 cm^3^·m^−2^·day^−1^·atm^−1^ for O_2_ and ~400–600 cm^3^·m^−2^·day^−1^·atm^−1^ for CO_2_) and low-density polyethylene (LDPE, ~700–900 cm^3^·m^−2^·day^−1^·atm^−1^ for O_2_ and ~1300–1600 cm^3^·m^−2^·day^−1^·atm^−1^ for CO_2_, according to the supplier’s technical datasheet).

Each experimental unit consisted of one bag containing approximately nine pear wedges (about 135 g in total), representing a homogeneous sample of minimally processed fruit. Minimally processed pears were evaluated at harvest and after 5, 10, and 15 days of storage at 0 °C and 95% RH. Four replicate bags (i.e., four experimental units) were analyzed per treatment and evaluation time.

### 3.3. Respiration Rate Determination

About 150 g of pear wedges were placed in an 820 mL hermetic glass container for respiration rate determination. The containers were provided with a silicone septum on top to avoid the headspace gas extraction (10 mL) by a plastic syringe after 1.5 h of closure. The samples were then injected into a gas chromatograph (Agilent Technologies, 7890B, Santa Clara, CA, USA) equipped with an injector, oven, and detector at 20, 60, and 200 °C, respectively. Measurements were carried out in 4 repetitions, and the values were expressed as mL CO_2_. kg^−1^.h^−1^.

### 3.4. Atmosphere Composition

The internal atmosphere composition was determined by a portable gas analyzer (Dansensor, Check Point, Ringsted, Denmark). The samples were extracted through silicone septum placed in each bag prior to determination. The values were expressed as percentages.

### 3.5. Flesh Firmness Measurement

Flesh firmness was measured using a texture analyzer (TA.XT Plus, Hamilton, MA, USA). Determinations were made on the central part of six pear wedges by repetition using a 3 mm diameter stem that penetrated 5 mm at 10 mm s^−1^. The values were expressed in N.

### 3.6. Color Determination

Measurements were carried out in the central part of 6 wedges per repetition and for evaluation. A tri-stimulus colorimeter (Precise Color Reader, TCR 200, Beijing, China) was used. The values were expressed as the CIELab* color system parameters, luminosity (L*), tone (h_ab_*) and saturation (C*).

The total color difference (ΔE) expresses the difference between the initial color of the pulp (time zero) and the color after storage of the pear slices. The total color difference (ΔE) was calculated according to the following formula:

∆E* = [(ΔL*)^2^ + (Δa*)^2^ + (Δb*)^2^]^1/2^

### 3.7. Polyphenol Oxidase Activity

Activity determination was carried out according to the methodology proposed by Sanchís et al. [50]. For this purpose, 15 g of pulp was homogenized (Scientz, XHF-D, Ningbo, China) with an extraction solution composed of McIlvaine buffer solution at pH 6.5 containing 1 M sodium chloride and 50 g kg^−1^ of polyvinylpyrrolidone for 2 min at 150,000 rpm. To obtain the supernatant used in the determination, the mixture was centrifuged at 12,000 rpm and 4 °C for 30 min (Thermo Scientific, Sorvall ST 16R, Dreieich, Germany). For the measurement of enzyme activity, 100 µL of the extract was placed in a quartz cuvette and 3 mL of a 0.05 M solution of 4-methyl-catechol was added and the change in absorbance was recorded (Thermo Genesys 10 S, New Brunswick, NJ, USA) at 420 nm every 5 s for a period of 2 min.

### 3.8. Total Polyphenols and Total Antioxidant Capacity

Before determination, 3 g of the frozen samples was homogenized (Scientz, XHF-D, Ningbo, China) with 5 mL of methanol 70% *v*/*v*. After that, it was kept in the dark for 1 h and then centrifuged (Thermo Scientific ST16R, Germany) at 4 °C, 10 min at 15,000× *g*. The supernatant was used to determine the total polyphenol content and antioxidant activity.

The total polyphenol content was determined by the methodology proposed by Singleton and Rossi [51] modified by González et al. [52]. The results were expressed as mg of gallic acid equivalent per g sample in fresh weight (GAE g^−1^ FW). All determinations were performed in triplicate.

Antioxidant activity was determined using two methodologies: the one that used 2,2 diphenyl-1-picryhydrazyl (DPPH) reactive following the method proposed by Brand-Williams et al. [53] and the indirect method of the antioxidant reduction of ferric ion (FRAP) proposed by Benzi and Strain [54]. Both were partially modified and described by González et al. [52]. The antioxidant concentration was expressed as mg of ascorbic acid equivalents per g of sample in fresh weight (mg AAE g^−1^ FW). All determinations were performed in triplicate.

### 3.9. Microbiological Growth

For microbiological evaluation, 10 g samples, placed in sterilized bags (Nasco, Whirl-Pak, Pleasant Prairie, WI, USA) with 90 mL of sterilized peptone water (Oxoid Limited, Basingstoke, UK), were manually homogenized. Aliquots of 1 mL for mesophilic, psychrophilic, and Enterobacteriaceae, and of 0.1 mL for molds and yeast were sown in discarded Petri dishes. Mesophiles, psychrophiles, and Enterobacteriaceae were sown by inclusion, while fungi and yeast were on the surface with a Drigalskiy loop. Serial dilutions were performed when necessary.

Mesophilic and psychrophilic were sown in count agar culture medium (Oxoid, Limited, Basingstoke, UK) and incubated at 37 °C for 24 h; mesophilic were incubated at 4 °C for 7 days. The enterobacteria were sworn into violet-red bile agar (Oxoid Limited, Basingstoke, UK) and incubated at 37 °C for 24 h. Fungi and yeast were sown on potato dextrose agar medium (Oxoid Limited, Basingstoke, UK) amended with streptomycin sulfate (0.2 g L^−1^) (Sigma-Aldrich, St. Louis, MO, USA). The plates were incubated for 7 days at 25 °C. The results were expressed as log units of colony-forming units per g of fresh weight (Log CFU g^−1^ FW).

### 3.10. Statistical Analysis

Statistical analyses were performed in Navure software (2.7.3) using R version 3.6.3 [55].

A factorial ANOVA was used to evaluate the effect of three factors: raw material storage conditions (harvest, 3 months in regular atmosphere; 3 and 6 months in controlled atmosphere), storage time of the minimally processed pear (0, 5, 10, and 15 days), and bag type (PP and LDPE), as well as their possible interactions. When significant differences were detected (*p* < 0.05), Tukey’s multiple comparison test was applied to determine differences between the levels within each factor. The results are presented as mean ± standard error of the mean.

Additionally, Pearson’s correlation analysis was performed to evaluate the relationships among the studied variables. Correlation coefficients (r) and significance levels (*p*-values) are reported.

## 4. Conclusions

The preservation conditions of the raw material strongly influenced the quality of fresh-cut pears during refrigerated storage. Storing whole pears under CA conditions for 3 or 6 months prior to processing was effective in preserving color, firmness, and functional quality (polyphenol content) while also reducing enzymatic browning (PPO activity) and respiration rate. Importantly, no significant quality differences were observed between 3 and 6 months of CA storage, suggesting that high-quality raw material can be maintained for up to 6 months. Although differences between packaging materials were not substantial, pears packaged in polypropylene (PP) showed a slight tendency toward better quality retention, possibly due to reduced oxygen permeability. However, further research is needed to confirm this effect or to identify alternative materials with improved performance.

Therefore, storing pears under CA conditions for up to 6 months appears to be a suitable strategy to ensure the quality of fresh-cut products.

## Figures and Tables

**Table 1:** Color parameters of pear wedges from different raw material origins (RA: regular atmosphere; CA: controlled atmosphere) stored for 15 days in different packaging material (PP: polypropylene, LDPE: low density polyethylene).

PP				
L*				
Treatment/time	0 d	5 d	10 d	15 d
Harvest	65.76 ± 0.90 Ab	64.98 ± 1.22 Bb	70.74 ± 0.66 Aa	59.27 ± 1.70 Bc
3 m RA	55.07 ± 1.11 Ba	57.91 ± 1.00 Ca	58.25 ± 1.02 Ca	54.70 ± 1.53 Ca
3 m CA	68.89 ± 0.71 Aa	65.91 ± 0.83 Ba	65.19 ± 1.90 Ba	66.21 ± 0.91 Aa
6 m CA	68.59 ± 0.54 Aa	70.62 ± 0.64 Aa	70.53 ± 0.64 Aa	67.32 ± 0.71 Aa
h*_ab_				
Harvest	93.31 ± 0.79 Aa	87.78 ± 0.83 Ab	90.39 ± 0.99 Aab	90.04 ± 0.58 Aab
3 m RA	92.45 ± 1.34 Aa	82.05 ± 0.86 Bc	85.65 ± 1.40 Bb	81.52 ± 1.13 Bc
3 m CA	87.06 ± 0.69 Ba	82.20 ± 0.65 Bb	77.79 ± 0.81 Cc	82.81 ± 0.94 Bb
6 m CA	88.79 ± 1.91 Ba	78.11 ± 2.48 Cb	84.35 ± 0.78 Bc	83.90 ± 0.84 Bc
C*				
Harvest	6.74 ± 0.18 Bb	7.01 ± 0.25 Cb	7.55 ± 0.22 Bb	14.66 ± 0.31 Aa
3 m RA	7.61 ± 0.25 Ba	7.82 ± 0.39 BCa	9.08 ± 0.26 Ba	8.69 ± 0.30 Ca
3 m CA	8.47 ± 0.24 ABc	10.76 ± 0.57 Ab	15.19 ± 1.23 Aa	11.18 ± 0.56 Bb
6 m CA	9.79 ± 0.24 Aa	9.02 ± 0.29 ABa	9.14 ± 0.26 Ba	10.05 ± 0.31 BCa
LDPE				
L*				
Harvest	63.46 ± 1.33 Ab	62.99 ± 1.38 Bb	70.08 ± 1.39 Aa	48.07 ± 4.07 Cc
3 m RA	56.44 ± 0.68 Ba	53.58 ± 1.10 Ca	52.98 ± 1.07 Ca	53.80 ± 1.15 Ba
3 m CA	67.24 ± 0.56 Aa	63.77 ± 0.71 Ba	64.44 ± 0.98 Ba	65.00 ± 0.61 Aa
6 m CA	66.70 ± 0.91 Aa	68.11 ± 0.55 Aa	67.69 ± 0.60 ABa	68.61 ± 0.74 Aa
h*_ab_				
Harvest	94.23 ± 0.97 Aa	91.32 ± 0.64 Aa	93.12 ± 0.92 Aa	91.55 ± 0.69 Aa
3 m RA	89.44 ± 0.86 Ba	82.11 ± 1.11 Bb	80.05 ± 0.98 Bb	80.75 ± 1.07 Cb
3 m CA	85.60 ± 0.49 Ca	80.52 ± 0.60 Bb	74.80 ± 0.98 Cc	81.21 ± 0.93 BCb
6 m CA	89.43 ± 0.79 Ba	73.61 ± 0.48 Cd	80.41 ± 0.93 Bc	84.72 ± 0.90 Bb
C*				
Harvest	7.04 ± 0.19 Ab	6.40 ± 0.18 Cb	6.97 ± 0.22 Cb	11.47 ± 1.07 ABa
3 m RA	8.30 ± 0.27 Aa	7.82 ± 0.38 BCa	9.05 ± 0.32 Ba	9.07 ± 0.33 Ca
3 m CA	8.84 ± 0.25 Ac	12.07 ± 0.57 Ab	20.80 ± 1.79 Aa	12.29 ± 0.77 Ab
6 m CA	8.89 ± 0.32 Aa	9.50 ± 0.22 Ba	10.12 ± 0.33 Ba	10.38 ± 0.29 BCa

Values are means (n = 18) ± standard error of the mean. Uppercase letters indicate differences between raw material origins, in each storage time, and lowercase letters indicate differences over storage time within each packaging type (Tukey’s test, *p* < 0.05).

## Data Availability

The data presented in this study are not publicly available but may be obtained from the corresponding author upon reasonable request.

## Abbreviations

The following abbreviations are used in this manuscript:

CA	Controlled Atmosphere
RA	Regular Atmosphere
MAP	Modified Atmosphere Packaging
MPF	Minimally Processed Fresh
PPO	Polyphenol Oxidase
ΔE	Total Color Difference
L*	Lightness
C*	Chroma
h_ab_*	Hue Angle
TP	Total Polyphenol
TAC	Total Antioxidant Capacity
DPPH	2,2-Diphenyl-1-picrylhydrazyl (Radical Scavenging Assay)
FRAP	Ferric Reducing Antioxidant Power
GAE	Gallic Acid Equivalents
AAE	Ascorbic Acid Equivalents
FW	Fresh Weight
CFUs	Colony-Forming Units
PP	Polypropylene
LDPE	Low-Density Polyethylene
